# [^68 ^Ga]Ga-FAPI-46 PET/CT for locoregional lymph node staging in urothelial carcinoma of the bladder prior to cystectomy: initial experiences from a pilot analysis

**DOI:** 10.1007/s00259-024-06595-z

**Published:** 2024-01-18

**Authors:** Lena M. Unterrainer, Lennert Eismann, Simon Lindner, Franz-Josef Gildehaus, Johannes Toms, Jozefina Casuscelli, Adrien Holzgreve, Sophie C. Kunte, Clemens C. Cyran, Paula Menold, Alexander Karl, Marcus Unterrainer, Stephan T. Ledderose, Christian G. Stief, Peter Bartenstein, Alexander Kretschmer, Gerald B. Schulz

**Affiliations:** 1 Department of Nuclear Medicine, LMU University Hospital, LMU Munich, Munich, Germany; 2 Ahmanson Translational Theranostics Division, Department of Molecular and Medical Pharmacology, David Geffen School of Medicine, UCLA, Los Angeles, USA; 3 Department of Urology, LMU University Hospital, LMU Munich, Munich, Germany; 4 Department of Radiology, LMU University Hospital, LMU Munich, Munich, Germany; 5 Department of Urology, Krankenhaus Barmherzige Brüder, Munich, Germany; 6 Die RADIOLOGIE, Munich, Germany; 7 Institute of Pathology, LMU Munich, Munich, Germany; 8 Janssen Research and Development, Los Angeles, USA

**Keywords:** Urothelial carcinoma, Cystectomy, FAP, FAPI, PET/CT imaging, Staging, Bladder cancer

## Abstract

**Introduction:**

[^68^ Ga]Ga-FAPI-46 PET/CT is a novel hybrid imaging method that previously showed additional diagnostic value in the assessment of distant urothelial carcinoma lesions. We hypothesized that patients with bladder cancer benefit from [^68^ Ga]Ga-FAPI-46 PET/CT prior to radical cystectomy for locoregional lymph node staging.

**Materials and methods:**

Eighteen patients underwent [^68^ Ga]Ga-FAPI-46 PET/CT for evaluation of lymph node (LN) status in predefined LN regions. Two hundred twenty-nine intraoperatively removed LN served as histopathological reference standard.

**Results:**

Urothelial carcinoma (UC) spread was found in ten LN in seven different regions (14.3%). Hereby, [^68^ Ga]Ga-FAPI-46 PET/CT was positive in four out of seven regions (57.1%) and showed significantly increased FAPI uptake compared to non-pathological regions. In the remaining three out of seven (42.9%) regions, [^68^ Ga]Ga-FAPI-46 PET/CT was rated negative since no pathological increased FAPI uptake was detected or the proximity of the urinary tract prevented a differentiation from physiological uptake. CT was inconspicuous in these three regions. In total, two FAP-positive LN regions were found without histopathological counterpart. Overall, sensitivity, specificity, positive predictive value, and negative predictive value were 57.1%, 95.2%, 66.7%, and 93.0% for PET imaging.

**Conclusion:**

In summary, this innovative [^68^ Ga]Ga-FAPI-46 PET/CT method showed high specificity and negative predictive value in patients with bladder UC with a future potential to optimize therapy planning.

**Supplementary Information:**

The online version contains supplementary material available at 10.1007/s00259-024-06595-z.

## Introduction

A relevant unmet need in patients with muscle-invasive urothelial carcinoma (UC) of the bladder is the evaluation of the locoregional lymph node status prior to a planned radical cystectomy (RC) as the clinical management and prognosis depend on the locoregional disease extent and the presence of lymph node (LN) metastases is one of the main risk factors for poor oncological outcome [1, 2]. Clinical to pathologic stage discrepancy is a relatively common finding in bladder cancer (BC) with a postoperative pathologic upstaging in a relevant portion of patients (20–25%) [3, 4] compared to the findings on conventional imaging (CT/MRI) prior to a RC.

As no advantage for locoregional LN staging could be observed for [^18^F]FDG-PET/CT compared to CT in the pre-RC setting [5] and [^18^F]FDG-PET/CT is not implemented as staging method prior RC in the current guidelines [6], an adequate preoperative staging with a novel hybrid imaging method might lead to an improved patient-individual surgery procedure and LN-region specific treatment. Given the potentially high morbidity accompanying radical lymphadenectomy, optimal treatment planning or even omission of specific LN regions is crucial.

[^68^ Ga]Ga-FAPI-46 PET/CT is a promising molecular imaging method, which targets the overexpression of fibroblast activation protein (FAP) in the cancer associated fibroblasts of the tumor microenvironment [7] and has been assessed in various tumor entities [8]. It could be shown that the FAP expression in UC correlates with tumor staging and favors tumor invasion in high-grade invasive UC [9]. We recently showed feasibility of [^68^ Ga]Ga-FAPI-46 PET/CT in a mixed pilot cohort of patients with advanced UC [10]. Given these promising results and the paucity of data for loco-regional LN staging prior to RC, we hypothesized that [^68^ Ga]Ga-FAPI-46 PET/CT could improve LN staging in this challenging clinical setting.

## Materials and methods

Eighteen patients (mean age 71.8 years, range 58–82 years) with muscle-invasive UC (lowest T:T2a) underwent [^68^ Ga]Ga-FAPI-46 PET/CT for evaluation of locoregional LN status prior to RC with pelvic lymph node dissection (PLND) and were included in the current analysis. Inclusion criteria encompassed (1) histopathologically proven high-grade UC of the bladder and (2) [^68^ Ga]Ga-FAPI-46 PET/CT less than 40 days before cystectomy. This analysis was performed in compliance with the principles of the Declaration of Helsinki and its subsequent amendments and retrospective analysis of data was approved by the institutional ethics board of the LMU Munich.

Following the regulations of the German Pharmaceuticals Act §13(2b), the labeling of the FAPI tracers was performed under the direct responsibility of the applying physician. FAPI-46 was provided by SOFIE (21000 Atlantic Blvd., Ste 730, Dulles, VA 20166). The radiolabeling of FAPI was performed as previously described [10]. Patients were instructed to empty the bladder and premedicated with furosemide (Furosemid-ratiopharm 20 mg/2 mL injection solution, ratiopharm GmbH, Ulm, Germany) if not contraindicated. PET/CT was performed, and images were reconstructed as previously described [10]. In case of a prior diagnostic CT, a low-dose CT was performed concomitant with the PET with a mean time of 21 (0–47) days between PET and CT. CT findings were directly correlated with the [^68^ Ga]Ga-FAPI-46 PET scan on a per-patient basis.

PET analyses were performed using a dedicated software package (Hermes Hybrid Viewer, Hermes Medical Solutions, Stockholm, Sweden). PET-positive nodes were identified by increased [^68^ Ga]Ga-FAPI-46 uptake above the background (SUV_max_/SUV_mean___background_; ratio 2:1) and compared to histopathological results. In regions with at least one histopathologically proven LN metastasis, the LN that showed the highest SUV_max_ and the respective short-axis diameter (SAD) were determined. In regions without LN metastases based on histopathology, the LN with the highest SUV_max_ and the corresponding SAD were likewise determined, if measurable. CT positive LN were defined by common criteria [11].

All patients underwent RC with PLND following previously described templates (1). Mean (min/max) period between RC and PET/CT scan was 12 (1–36) days. Patients were monitored between tracer injection and end of PET/CT scan; no safety concerns were reported.

LNs were classified by anatomical region as follows: common iliac, internal iliac, external iliac, and obturatoric or pelvic (each left/right, respectively). Histopathological results were compared to preoperative [^68^ Ga]Ga-FAPI-46 PET/CT and diagnostic CT.

## Results

**Fig. 1:**
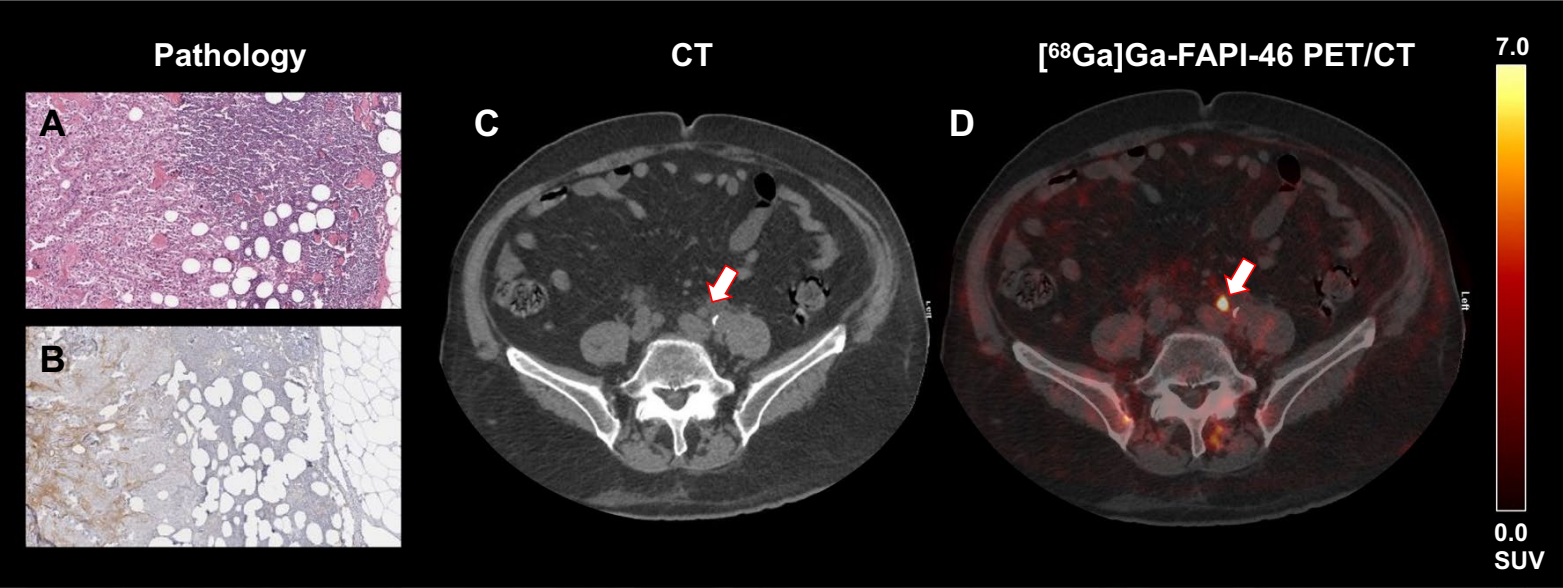
Histopathological proven LN metastasis of a left pelvic LN (**A**). FAP staining confirmed increased FAP expression in this LN metastases (**B**). On CT, the LN did not meet the criteria of a pathological LN in the CT component (SAD 7.0 mm; arrow) (**C**). [^68^ Ga]Ga-FAPI-46 PET/CT showed a highly increased FAPI uptake of this lymph node (arrow, **D**)

In the included 18 patients, 229 histopathological LN samples in 49 LN regions were analyzed. In six of the 18 patients, seven regions showed histopathologically proven LN metastases (14.3%, positive LN ratio: 10/229, 4.4%) (Fig. 1). Diagnostic accuracy of [^68^ Ga]Ga-FAPI-46 PET/CT and CT is presented in Table 1. The scans were read by two experienced nuclear medicine physicians. [^68^ Ga]Ga-FAPI-46 PET/CT revealed positive pathological uptake in four out of seven regions [57.1%; median SUV_max_ 12.1 (7.6–27.2) / median SAD 0.8 (0.3–1.6) mm]. In the remaining 42 regions, there was either no lesion with increased FAPI-uptake or LN were not evaluable due to increased urinary activity. Notably, there was also no suspicious LN in each of these regions in the CT scan.

**Table 1:** Diagnostic accuracy

	[^68^ Ga]Ga-FAPI-46 PET	Contrast-enhanced CT
True positive	4	2
False positive	2	1
True negative	40	41
False negative	3	5
Sensitivity	57.1%	28.6%
Specificity	95.2%	97.6%
Positive predictive value	66.7%	66.7%
Negative predictive value	93.0%	89.1%

In histopathological-negative regions, median SUV_max_ [1.1 (0.3–2.5) versus 12.1 (7.6–27.2)] and median SAD [1.2 (0.3 –2.5) versus 0.7 mm (0.4–1.2)] was significantly lower than in histopathologically positive LN regions (SUV_max_: *p* < 0.01; SAD: *p* = 0.031). Two patients were rated positive in the pelvic area in the [^68^ Ga]Ga-FAPI-46 PET/CT without positive findings in the histopathological work-up. One patient showed increased FAPI-uptake in the pelvic region (SUV_max_ 8/SAD 0.7 mm); the other patient presented with a FAPI positive LN in the common iliac region which was also rated suspicious in CT scan (SUV_max_ 30.6/SAD 1.2 cm). As shown in Table 1, sensitivity, specificity, positive predictive value (PPV), and negative predictive value (NPV) for detection of affected LN regions were 57.1%, 95.2%, 66.7%, and 93.0% for PET criteria and 28.6%, 97.6%, 66.7%, and 89.1% for CT.

## Discussion

This retrospective analysis is the first of its kind to assess the use of [^68^ Ga]Ga-FAPI-46 PET/CT in localized UC prior RC. In this patient cohort, [^68^ Ga]Ga-FAPI-46 PET/CT shows a high specificity with a NPV of 93.0%. Furthermore, [^68^ Ga]Ga-FAPI-46 PET showed an increased sensitivity compared to diagnostic CT alone, although the rate of 57.1% is still only moderate. This low rate may be interpreted in the context of the low number of histopathologically confirmed LN metastases in our pilot cohort. In three out of seven regions with histopathologically proven LN metastases, it was not possible to delineate PET- and CT-positive LN. In CT, this is likely due to size of the LN. For PET assessments, the relatively high urine activity of [^68^ Ga]Ga-FAPI-46 and the spatial volume effect were identified as a major contributor to false-negative findings. Here, it can be hypothesized that another labeled FAPI radioligand such as [^18^F]-FAPI-74 might be superior in an UC cohort due to diverging hepatobiliary and renal excretion of the ligand.

Two LN in two different regions were rated falsely positive in the PET scan. Here, two scenarios can be hypothesized: First, the increased FAPI uptake could represent a LN metastasis that was missed and not dissected during the PLND. As this analysis was retrospectively, there was no standardized PLND following a prospective trial protocol. Alternatively, one can speculate about a reactive inflammatory process since FAPI PET has been shown to be positive in inflammatory mechanisms even if in the respective lymph node regions, there was no inflammatory process described in the histopathological report [12].

This short communication is limited by its small cohort size as well as by its retrospective design in which a systematic intraoperative PLND could not be performed. Future systematic studies are warranted to evaluate better this novel approach for evaluating locoregional LN metastases prior RC in UC patients.

## Conclusion

This pilot analysis is to our knowledge the first study that assessed the potential benefit of [^68^ Ga]Ga-FAPI-46 PET/CT in high-grade UC of the bladder prior cystectomy: Against the high NPV and specificity in this cohort, it can be hypothesized that [^68^ Ga]Ga-FAPI-46 PET/CT might have a future potential to optimize therapy planning, i.e., in omitting non-affected locoregional lymph node regions.

### Supplementary Information

Below is the link to the electronic supplementary material.

Supplementary file1 (PPTX 36 KB)
