# Supplementary material for: [68 Ga]Ga-FAPI-46 PET/CT for locoregional lymph node staging in urothelial carcinoma of the bladder prior to cystectomy: initial experiences from a pilot analysis
Source: Eur J Nucl Med Mol Imaging. 2024 Jan 18;51(6):1786–9. doi: 10.1007/s00259-024-06595-z (PMC11043110; doi:10.1007/s00259-024-06595-z)
Supplement: Supplementary file 1 — Supplementary file1 (PPTX 36 KB) [file 259_2024_6595_MOESM1_ESM.pptx]

## Slide 1
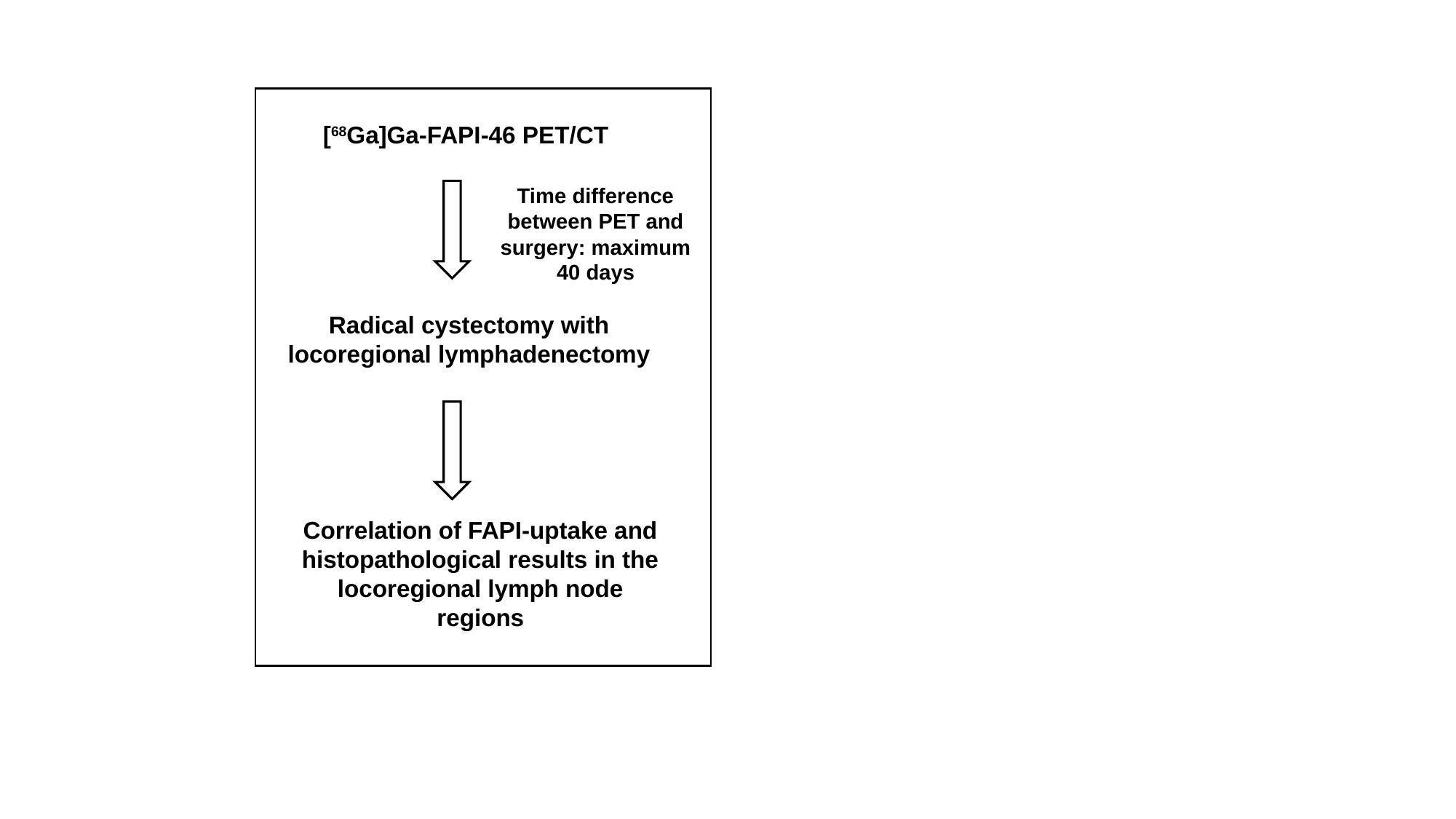

[68Ga]Ga-FAPI-46 PET/CT
Time difference between PET and surgery: maximum 40 days
Radical cystectomy with locoregional lymphadenectomy
Correlation of FAPI-uptake and histopathological results in the locoregional lymph node regions
